# A daily diary investigation of cannabis use and its diet and exercise correlates

**DOI:** 10.3389/fpsyg.2023.1217144

**Published:** 2023-08-04

**Authors:** Laurel P. Gibson, Carillon J. Skrzynski, Gregory R. Giordano, Angela D. Bryan

**Affiliations:** Department of Psychology and Neuroscience, University of Colorado Boulder, Boulder, CO, United States

**Keywords:** marijuana, delta-9-tetrahydrocannabinol, cannabidiol, health, physical activity, eating behavior, diary study

## Abstract

**Background:**

The increasing availability of legal-market cannabis products has raised many questions about potential harms and benefits of increased use. In particular, concerns have been raised about the possible negative impact of cannabis use on behavioral determinants of obesity and chronic disease, including diet and exercise. However, previous research is mixed and has largely relied on cross-sectional survey data and coarse measurements of cannabis use, underscoring the need for more rigorous research designs.

**Purpose:**

The present study utilized longitudinal daily diary data to assess whether exercise and diet patterns differed between cannabis users and non-users and, within cannabis users, whether legal-market cannabis use, diet, and exercise covaried within individuals across time and based on cannabinoid content.

**Methods:**

A sample of 98 participants (77 cannabis users, 21 non-users) completed a baseline appointment and a 30-day daily diary study assessing their daily cannabis use, diet, and exercise. Cannabis users were quasi-randomly assigned to use either a THC-dominant flower product (*n* = 36) or a CBD-containing flower product (*n* = 41) *ad libitum* over the course of the daily diary study. Participants were between the ages of 21 and 41 (*M* = 29.28) and were majority male (61.2%).

**Results:**

At baseline, there were no differences in BMI or exercise behavior between users and non-users. Likelihood of exercising and exercise minutes per day over the 30-day period also did not differ between users and non-users, nor did these outcomes differ on cannabis use vs. non-use days among cannabis using participants. In contrast, there was some evidence for a relationship between cannabis use and dietary measures. At baseline, non-users scored higher on the Healthy Eating Index than users. Daily data also indicated that users consumed marginally more salty snacks and fast food per day relative to non-users, and users consumed more fruits/vegetables and marginally more salty snacks on cannabis use days vs. non-use days. Interestingly, among users, no associations were dependent on the cannabinoid content of their assigned product.

**Conclusion:**

Findings suggest little association between cannabis use and exercise but underscore the need for further research on how cannabis use may impact dietary patterns. Future research should examine the impact of cannabis on non-behavioral pathways to obesity and chronic disease (e.g., metabolism).

## 1. Introduction

Cannabis legalization has gained traction in recent years, with the majority of US States having legalized cannabis in some form (Defense Information Systems Agency, 2022). In tandem with increased legalization has come small but steady increases in cannabis use, with almost 19% of individuals 12 years of age and older reporting past year use in 2021 (Substance Abuse and Mental Health Services Administration, 2021) compared to 17.5% in 2019 and 15% in 2017 (Substance Abuse and Mental Health Services Administration, 2019). At this point, the public health implications of cannabis legalization are only beginning to be understood, and more information regarding the potential benefits and harms of use is needed.

One major concern is how increased access to and use of cannabis may negatively affect lifestyle choices like physical activity and diet, which may have downstream influences on public health (e.g., obesity, type 2 diabetes, heart disease, certain cancers). Indeed, there is evidence that cannabis use is associated with increased caloric intake broadly (Smit and Crespo, 2001; Rodondi et al., 2006; Kruger et al., 2019) as well as increased consumption of unhealthy food specifically (Smit and Crespo, 2001; Kruger et al., 2019; Gelfand and Tangney, 2021; Romano et al., 2022). Additionally, public perception suggests that cannabis use is associated with less physical activity and greater sedentary behavior (c.f., “couch-lock”), and there is some empirical support for this perception (Vidot et al., 2017; West et al., 2020).

Paradoxically, several studies have found associations between cannabis use and *beneficial* health behavior and outcomes (Korn et al., 2018; York Williams et al., 2020; Ong et al., 2021; Smith et al., 2021). For example, a large, nationally representative sample found that frequent cannabis users engaged in more physical activity as measured by accelerometry than non-user while light users had greater odds of self-reporting physical activity (yes/no response to past month engagement in moderate to vigorous activities) relative to non-current users (Ong et al., 2021). Similarly, other studies show that compared to non-users, cannabis users have lower body mass indexes (BMIs; Smit and Crespo, 2001; Rodondi et al., 2006; Le Strat and Le Foll, 2011; Korn et al., 2018; Ross et al., 2020; York Williams et al., 2020), a lower prevalence of being overweight or obese (Hayatbakhsh et al., 2010; Le Strat and Le Foll, 2011), and a decreased likelihood of diabetes (Imtiaz and Rehm, 2018).

Given these conflicting findings, a more nuanced approach to understanding potential associations between cannabis use and health behaviors like diet and exercise is warranted. Much of the existing data has been collected through large retrospective or cross-sectional epidemiological surveys and has utilized coarse measurements of cannabis including categorizing use and user status into “no use/non-user” vs. “any use/user” over prolonged periods of time (e.g., lifetime or past year; Cousijn et al., 2018; Geissler et al., 2020; Bidwell et al., 2021). While the data generated from such studies have certainly been informative, the methodologies utilized preclude making any causal statements or even, in some cases, establishing temporal precedence. Additionally, operationalizing cannabis use as broad categories (user/non-user) over lengthy time periods may obscure fine-grained relationships between cannabis use and health behavior occurring acutely. Such categorizations also provide no information regarding how these relationships may vary based on exogenous cannabinoid content, specifically Δ9-tetrahydrocannabinol (THC) and cannabidiol (CBD) levels in cannabis products. However, this information would be useful as there is emerging evidence that the relative ratios of CBD and THC in cannabis products have differential effects on both biological and behavioral processes relevant to health (Bhattacharyya et al., 2010; Morgan et al., 2010; Englund et al., 2013; Gibson et al., 2022).

Finally, existing studies may also suffer from third-variable confounders based on between-subjects factors (e.g., differences in personality or demographic factors between cannabis users and non-users which may be associated with both use and health behavior). In contrast, study designs that implement more precise assessments, such as daily diary studies which allow researchers to model associations within persons over time, can help rule out this possibility. Likewise, although federal regulations generally preclude randomized controlled trials in legal-market cannabis research (Hagerty et al., 2015; Hutchison et al., 2019), methods in which participants are quasi-randomly assigned to *ad libitum* use of different products with varying cannabinoid content can help address potential confounds, help support causal claims, and shed greater light on how THC vs. CBD relate to health. Overall, work investigating how cannabis use generally, and cannabinoid content specifically, relates to diet and exercise engaged in at a daily level, within individuals over time, and utilizing random assignment whenever possible, is needed to provide a more nuanced understanding of the potential health benefits or harms of cannabis use.

The data herein are part of a larger longitudinal study of the impact of cannabis use and cannabinoid function on biomarkers associated with inflammation and insulin regulation (NCT04114903). As part of that study, daily diary data were collected to examine differences between cannabis users and non-users on measures of physical activity, diet, and BMI as well as compare individuals assigned to use a predominantly THC product vs. a product containing CBD on these same measures. First, to replicate prior work, we examined baseline differences in physical activity, diet, and BMI by user status (i.e., cannabis users vs. non-users). Next, using daily data collected over a 30-day period, we examined differences in daily exercise and diet by both user status (i.e., comparisons between users and non-users) and condition assignment (i.e., comparisons across the two user groups and non-users). Lastly, using daily data, we examined longitudinal, day-level associations between daily cannabis use, exercise, and diet, as well as whether these associations were moderated by user group (i.e., THC vs. CBD). Based on prior literature, we hypothesized that cannabis users would have lower BMI, engage in more physical activity, and have poorer diet quality compared to non-users at baseline. Given the exploratory nature of the daily associations between cannabis use and exercise/diet behavior and whether any of these associations varied by cannabinoid content, we did not have specific hypotheses for these effects.

## 2. Methods

### 2.1. Participants

This study was approved by the University of Colorado Boulder Institutional Review Board and was carried out in accordance with the Declaration of Helsinki. Participants were compensated in cash for completion of the baseline ($120) and received $0.50 in Amazon gift card credit for completion of each daily diary report, as well as an additional $10 credit for completing at least 23 of the 30 possible daily diary reports.

Participants were recruited from the greater Boulder-Denver area using social media posts and mailed flyers. Participants were screened for eligibility via an online survey or over the phone by trained research staff. Participants were eligible to participate if they met the following inclusion criteria: (1) age 21–40 years, (2) no known autoimmune disease (e.g., HIV), (3) no acute illness (e.g., influenza), (4) no daily tobacco use, (5) no heavy drinking as defined by an Alcohol Use Disorders Identification Test (AUDIT) score greater than 8 at the time of screening (Saunders et al., 1993), (6) no illicit drug use in the past 90 days (confirmed with a urine toxicology screen), (7) not currently using medications for glucose lowering, immunosuppression, or anti-inflammation, (8) no diagnosis of diabetes, (9) no current or history of a psychotic or bipolar disorder, (10) not currently using anti-psychotics or medications to treat bipolar disorder, (11) weight stable (less than 12 pound fluctuation in the last 6 months), (12) no blood donation in the 8 weeks prior to screening or intention to donate blood in the 8 weeks after completing the study, (13) have a fasting blood glucose ≥55 mg/dL and ≤ 126 mg/dL at the baseline appointment, (14) no diagnosis of chronic asthma, chronic bronchitis, or chronic obstructive pulmonary disease, (15) not on a carbohydrate restricted diet, (16) not pregnant (verified via pregnancy test), nursing, or trying to become pregnant, (17) have been a regular (at least weekly) cannabis user for at least a year (cannabis user group only), (18) use cannabis at least 8 days per month (cannabis user group only), and (19) have not used cannabis in the past year (non-user group only).

Changes to eligibility criteria were made after study commencement. The threshold for being considered weight stable (criterion #10) was increased from 5 pounds to 12 pounds to enhance recruitment. The exclusion of individuals with a chronic respiratory disease (criterion #13) was added to enhance safety as this study involves the use of inhaled cannabis flower. The exclusion of individuals on carbohydrate restricted diets (criterion #14) was added because of the insulin sensitizing effects of these diets (O’Neill, 2020; Yuan et al., 2020). All study data were collected and managed using REDCap electronic capture tools hosted at the Colorado Clinical and Translational Sciences Institute (Harris et al., 2009).

### 2.2. Procedures

#### 2.2.1. Baseline appointment

Participants were instructed to not use alcohol or cannabis for 24 h and to fast (no food or drink other than water) for 12 h prior to the baseline appointment. After providing informed consent, participants completed a breathalyzer test to ensure they had no detectable level of blood alcohol. Participants were screened via urinalysis for pregnancy (female participants only) and the presence of sedatives, cocaine, opiates, and amphetamines. Participants completed questionnaires of demographics and physical activity. Trained research staff conducted physical activity and dietary recall interviews, as well as measured participant height and weight.

At the end of the baseline appointment, participants in the cannabis user group were instructed to purchase one of three chemovars of cannabis flower, each with a different ratio of THC to CBD: THC-dominant, THC + CBD, or CBD-dominant. Regulations at both the federal level (National Institutes of Health) as well as the local level (Institutional Review Board) precluded us from utilizing true random assignment to a chemovar. Instead, participants completed a quasi-randomized selection process that included rolling a die where each number on the die corresponded to one of the chemovars with an equal chance of rolling each chemovar. After rolling, participants were told which chemovar group they were assigned to and could then accept that assignment or choose another chemovar. Cannabis using participants included in the final analytic sample accepted the chemovar assigned by the die roll 63.6% of the time (*Note:* 21 participants rolled the CBD chemovar. 5 accepted this assignment, 8 switched to the THC chemovar, and 8 switched to the THC + CBD chemovar. Thirty four participants rolled the THC chemovar. 25 accepted this assignment, and 9 switched to the THC + CBD chemovar. Twenty two participants rolled the THC + CBD chemovar. 19 accepted this assignment, and 3 switched to the THC chemovar).

Once product assignment was complete, participants in the cannabis user group were instructed to purchase enough of their product for 30 days of use from a local dispensary and to not use any other cannabis products during this time. The State of Colorado requires THC and CBD potencies to be listed on cannabis product labels, meaning that while the research staff responsible for collecting data were blind to condition, the participants were not and legally cannot be. Participants in the non-user group were instructed to continue their usual habits.

#### 2.2.2. Daily diary reports

For each of the 30 days following the baseline appointment, participants reported their cannabis use, exercise, and diet via an online survey, the link to which was emailed to them.

### 2.3. Baseline measures

#### 2.3.1. Demographics

Participants self-reported gender, age, sexual orientation, education, employment, race, and ethnicity at the baseline appointment.

#### 2.3.2. Substance use history

A 30-day Timeline Followback (TLFB; Sobell and Sobell, 1992) was administered via an online interactive calendar at the baseline appointment to collect information about the use of alcohol, cannabis, and tobacco (Martin-Willett et al., 2020). Participants also self-reported their age of first cannabis use at the baseline appointment.

#### 2.3.3. BMI

Height and weight were measured using a digital scale with a height rod (Tanita WB-800H; Tanita Corporation, Tokyo, Japan) at the baseline appointment to calculate BMI.

#### 2.3.4. Baseline physical activity

The Stanford 7-day Physical Activity Recall (PAR; Blair et al., 1985) was completed at the baseline appointment. The PAR is an interviewer led questionnaire where participants report their total days of exercise, minutes of moderate exercise, and minutes of hard exercise during the past seven days.

#### 2.3.5. Baseline nutrition

Baseline dietary intake data were collected using the Nutrition Data System for Research (NDSR; Schakel et al., 1997) software versions 2019, 2020, and 2021, developed by the Nutrition Coordinating Center (NCC), University of Minnesota, Minneapolis, MN. Final calculations of total calories consumed and Healthy Eating Index (HEI) scores were completed using NDSR version 2022.

### 2.4. Daily diary measures

#### 2.4.1. Cannabis use

Daily cannabis use was assessed using a single question: “*how many grams of cannabis did you use yesterday?.”* Visual stimuli of cannabis flower (e.g., using pictures of 0.5 g and 0.25 g relative to a quarter and credit card) were presented to aid participants in more accurately estimating amounts of cannabis used. To create a binary cannabis use day variable, we coded 0 grams of cannabis as a non-use day and anything greater than 0 grams of cannabis as a cannabis use day.

#### 2.4.2. Exercise

Daily exercise was assessed using a single question: “*how many minutes did you exercise yesterday?*.” Like with cannabis use, a binary exercise variable was created by coding 0 min of exercise as a non-exercise day and anything greater than 0 min of exercise as an exercise day.

#### 2.4.3. Diet

Diet was assessed with five questions asking for the number of servings consumed on the previous day (e.g., “*How many servings of fruits/vegetables did you eat yesterday?*”) of fruits and vegetables, salty snacks (e.g., chips), sweet treats (e.g., cookies, candy), fast food (e.g., pizza, fries), and sugary drinks (e.g., soda, juice). *Note:* Items assessing consumption of salty snacks, sweet treats, fast food, and sugary drinks were added midway through the study. As such, data on consumption of salty snacks, sweet treats, fast food, and sugary drinks is only available for 15.3% of participants (*n* = 15).

### 2.5. Planned analyses

We first examined baseline differences in exercise, diet, and BMI by user status (non-user vs. user) by conducting a series of independent-samples *t*-tests (one for each of the dependent variables). We next examined differences in exercise and diet across the 30 days by user status (non-user vs. user). To do so, we conducted a series of multilevel models (one for each of the dependent variables) estimating random intercepts to determine whether cannabis users and non-users were different in terms of their exercise minutes, likelihood of exercising, and servings of fruits and vegetables, salty snacks, sweet treats, fast food, and sugary drinks.

Additionally, we examined differences in exercise and diet across the 30 days by chemovar condition assignment (i.e., comparisons across the user groups and non-users). Given the small number of participants who elected to use a CBD-dominant product (*n* = 5), we pooled participants who used a CBD-dominant product with participants who used a THC + CBD product to create an “any CBD” user group, resulting in three comparison groups: non-users, users assigned to a THC-dominant product, and users assigned to a product with CBD. We then conducted a series of multilevel models (one for each of the dependent variables) estimating random intercepts to determine whether non-users, users assigned to a THC-dominant product, and users assigned to a product with CBD were different in terms of any of the outcomes. In each model, we included a set of two orthogonal contrast codes to test for group differences. For each outcome of interest (e.g., exercise minutes), we ran three models varying the set of orthogonal contrast codes to test for relevant group differences: THC vs. any CBD, THC vs. non-user, and any CBD vs. non-user.

The last aim of the study was to examine day-to-day associations between cannabis use, exercise, and diet among cannabis users. For these analyses, we limited the analytic sample to cannabis users only (*n* = 77) and conducted a series of multilevel models estimating random intercepts and slopes for participant. In each model, daily cannabis use (non-use day vs. use day) was included as a predictor to examine whether exercise and diet varied on days when participants used cannabis vs. days when they did not use cannabis. User group (THC vs. any CBD) and the user group X daily cannabis use interaction term were included in each model to examine whether daily associations between cannabis, exercise, and diet were moderated by user group.

All analyses were conducted in R version 4.1.2.^1^ Multilevel models were estimated using the lme4 package version 1.1–25 (Bates et al., 2015) which implements maximum likelihood (ML) estimation.

## 3. Results

One hundred thirty participants were recruited for the study, of whom 98 completed at least 22 daily surveys. Thus, our final sample consisted of 98 participants (men = 60, women = 37, non-binary = 1), of whom 77 were cannabis users (THC = 36, any CBD = 41) and 21 were non-users. Participants who completed ≥22 daily surveys were less likely to identify as Hispanic (*p* = 0.02) and reported engaging in fewer minutes of hard exercise (*p* = 0.03), drinking on fewer days (*p* = 0.04), and initiating cannabis use at an earlier age (*p* = 0.05) compared to participants who completed <22 daily surveys. No other significant differences in demographics or baseline characteristics emerged between participants who completed ≥22 daily surveys and participants who completed <22 daily surveys. Demographics and substance use characteristics overall and by user status and cannabis condition are presented in Table 1. Participants were, on average, 29.98 years old [*standard deviation (SD)* = 5.60; range = 21–41], and the majority were non-Hispanic White (82.8%). Although there was a higher proportion of male participants in the cannabis user group compared to the non-user group, no other significant differences in demographics emerged between users and non-users. There were no significant differences in demographics by cannabis condition.

**Table 1:** Baseline demographics and substance use patterns overall and by user group, and, among cannabis users, by cannabis condition.

	Overall	Non-users	Users		Users (THC)	Users (CBD)	
	*N* = 98	*N* = 21	*N* = *77*	*p*-value	*n* = 36	*n* = 41	*p*-value
*Demographics*							
Gender (Male)	61.2%	38.10%	67.50%	**0.01**	72.20%	63.40%	0.56
Age	29.98 (*5.60*)	29.95 (*7.18*)	29.99 (*5.14*)	0.98	29.5 (*4.60*)	30.41 (*5.60*)	0.44
Sexual orientation (% Heterosexual)	72.7%	66.7%	75.0%	0.66	66.70%	85.70%	0.58
Education (% Bachelor’s or Above)	69.4%	71.4%	68.8%	0.76	61.10%	75.60%	0.38
Employment (% Employed Full-Time)	66.30%	66.7%	66.2%	0.83	66.70%	65.90%	0.96
Race (% white)	87.80%	81.8%	88.3%	0.60	88.90%	87.80%	0.31
Ethnicity (% non-hispanic)	14.30%	95.2%	83.1%	0.29	88.90%	78.00%	0.34
*Substance use patterns*							
Cannabis flower use days	13.35 (*11.71*)	0.00 (*0.00*)	16.82 (*10.68*)	**< 0.001**	18.64 (*9.14*)	15.22 (*11.74*)	0.16
Cannabis edible use days	1.29 (*3.60*)	0.00 (*0.00*)	1.62 (*3.98*)	**< 0.001**	0.61 (*1.38*)	2.51 (*5.17*)	**0.04**
Cannabis concentrate use days	4.78 (*8.59*)	0.00 (*0.00*)	6.03 (*9.25*)	**< 0.001**	4.03 (*8.22*)	7.78 (*9.83*)	0.08
Alcohol use days	6.53 (*6.78*)	4.45 (*5.6*)	7.06 (*6.99*)	0.13	7.14 (*7.89*)	7.00 (*6.18*)	0.93
Tobacco use days	0.89 (*4.09*)	0.00 (*0.00*)	1.12 (*4.57*)	0.28	1.53 (*5.94*)	0.76 (*2.91*)	0.46
Age at first cannabis use	16.93 (*3.15*)	18.09 (*3.81*)	16.77 (*3.03*)	0.19	16.56 (*2.96*)	16.95 (*3.12*)	0.57

Bolded values indicate significance at *p* < 0.05.

### 3.1. Cannabis use, exercise, and diet at baseline

Exercise, diet, and BMI overall and by user group are presented in Table 2. The average BMI of the sample fell within the healthy weight range (i.e., 18.5 to <25.0; Centers for Disease Control and Prevention, 2022) and, on average, participants exceeded national physical activity guidelines (i.e., ≥ 150 min moderate exercise, or ≥ 75 min vigorous exercise per week; American College of Sports Medicine, 2021). Total scores on the HEI were slightly below the national average (i.e., 58; USDA, 2022) and, on average, participants did not meet national fruit and vegetable intake recommendations (i.e., ≥ 1.5 cup-equivalents of fruits and ≥ 2 cup-equivalents of vegetables; Centers for Disease Control and Prevention, 2022). Participants’ added sugar scores on the HEI indicated that their added sugar intake was less than 10% of their total daily calories, in line with national recommendations (Centers for Disease Control and Prevention, 2021). Few significant differences in exercise, diet, and BMI emerged between cannabis users and non-users at baseline. At baseline, non-users and users did not significantly differ in terms of total days of exercise, minutes of moderate exercise, or minutes of hard exercise reported over the previous 7 days. Further, although non-users scored higher on the HEI than cannabis users, non-users and users did not significantly differ in terms of total calories, fruits, vegetables, and added sugars consumed the previous day. Additionally, BMI was not significantly different between cannabis users and non-users.

**Table 2:** Baseline BMI, exercise, and diet overall and by user group.

	Overall	Non-Users	Users	
	*N* = 98	*N* = 21	*N* = *77*	*p*-value
*BMI*	24.29 (*5.20*)	23.87 (*2.42*)	24.41 (*5.73*)	0.68
*Physical activity recall (PAR)*				
Total days of exercise	4.86 (*1.92*)	5.24 (*1.84*)	4.75 (*1.93*)	0.31
Minutes of moderate exercise	336.76 (*455.67*)	363.57 (*446.57*)	329.44 (*460.74*)	0.76
Minutes of hard exercise	175.41 (*336.36*)	98.95 (*131.46*)	196.26 (*371.21*)	0.24
*Healthy eating index (HEI)*				
Total score	50.63 (*14.52*)	57.31 (*11.77*)	48.81 (*14.73*)	**0.02**
Total fruit (Cup-Equivalents)	0.49 (0.75)	1.82 (*1.66*)	1.24 (*1.71*)	0.25
Total vegetable (Cup-Equivalents)	1.78 (1.44)	3.03 (*1.90*)	3.26 (*1.66*)	0.34
Added sugar score	8.17 (*2.72*)	8.02 (*2.46*)	8.21 (*2.80*)	0.78
*Total calories consumed*	1966.66 (*801.24*)	2179.64 (*846.31*)	1908.57 (*784.18*)	0.17
*Carbohydrates (grams)*	219.99 (*100.62*)	253.25 (*113.20*)	210.92 (*95.71*)	0.09
*Fat (grams)*	88.77 (*48.76*)	96.76 (*47.50*)	86.59 (*49.17*)	0.40
*Sodium (milligrams)*	3555.09 (*1724.56*)	3105.33 (*1355.34*)	3677.75 (*1800.37*)	0.18
*Protein (grams)*	80.22 (*40.69*)	85.23 (*44.14*)	78.85 (*39.89*)	0.53

Bolded values indicate significance at *p* < 0.05.

Table 3 presents zero-order correlations between cannabis use patterns, exercise, diet, and BMI at baseline among the subgroup of cannabis users (*n* = 77; see Supplementary Table 1 for zero-order correlations across the two user groups). Few correlations between cannabis use patterns and diet/exercise emerged; however, more cannabis flower use days were associated with lower BMI, more edible use days were associated with consuming more calories and more fat, and more cannabis concentrate use days were associated with consuming more fruits. Additionally, initiating cannabis use later in life was associated with more minutes of moderate exercise.

**Table 3:** Bivariate correlations between baseline BMI, exercise, diet, and cannabis use variables among cannabis using participants (*N* = 77).

	1	2	3	4	5	6	7	8	9	10	11	12	13	14	15	16
1. BMI																
2. Total days of exercise	0.09															
3. Minutes of moderate exercise	0.04	**0.31**														
4. Minutes of hard exercise	−0.11	**0.27**	−0.13													
5. Total calories consumed	0.00	0.18	0.03	0.16												
6. Carbohydrates consumed	−0.13	0.08	−0.03	−0.17	**0.74**											
7. Fat consumed	0.06	0.15	0.07	**0.30**	**0.89**	**0.38**										
8. Sodium consumed	0.10	**0.25**	−0.02	**0.37**	**0.79**	**0.31**	**0.79**									
9. Protein consumed	0.03	0.03	0.04	0.18	**0.68**	**0.43**	**0.65**	**0.55**								
10. HEI total score	−0.13	0.06	−0.07	−0.09	0.01	0.07	0.00	0.02	−0.22							
11. HEI total fruit	−0.09	−0.21	−0.09	−0.16	−0.22	0.01	**−0.29**	−0.23	−0.35	**0.40**						
12. HEI total vegetable	0.00	0.20	−0.06	−0.02	0.09	0.07	0.09	0.09	0.04	**0.37**	−0.15					
13. HEI added sugar score	0.16	0.11	−0.03	0.19	0.02	**−0.29**	0.21	**0.26**	0.08	**0.36**	−0.03	**0.39**				
14. Cannabis flower use days	**−0.25**	0.05	0.11	−0.09	−0.12	−0.03	−0.13	−0.12	−0.19	0.16	0.03	0.04	0.03			
15. Cannabis edible use days	0.12	−0.01	−0.04	0.15	**0.23**	0.04	**0.35**	0.16	0.10	−0.01	−0.03	0.17	0.10	−0.06		
16. Cannabis concentrate use days	0.14	−0.12	0.03	0.02	−0.02	−0.08	0.01	0.05	−0.03	−0.02	**0.26**	−0.16	−0.15	**−0.49**	0.09	
17. Age at first cannabis use	−0.02	0.21	**−0.30**	0.20	0.05	0.06	−0.01	0.14	0.13	0.19	−0.04	0.08	0.11	−0.21	−0.12	0.02

Bolded values indicate significance at *p* < 0.05.

### 3.2. Cannabis use and exercise across the 30-day daily diary study

Across the 30-day daily diary study, participants exercised for an average of 41.55 min per day (*B* = 41.55, *standard error (SE)* = 3.14, *p* < 0.001) and reported engaging in exercise on 75% of the days in which they completed daily surveys. Likelihood of exercise (*odds ratio (OR)* = 1.11, 95% *confidence interval* (CI) [0.54, 2.27], *p* = 0.78) and average minutes of exercise per day (*B* = −5.19, *SE* = 7.66, *p* = 0.50) did not differ between cannabis users and non-users. Additionally, there were no significant differences in likelihood or minutes of exercise between non-users, users assigned to use THC-dominant products, and users assigned to use products with CBD (*p*s > 0.36).

Among cannabis users, likelihood of exercising (*OR* = 1.40, 95% CI [0.83, 2.34], *p* = 0.21) and minutes of exercise (B = −1.38, SE = 4.11, *p* = 0.74) did not differ on days when participants used cannabis vs. days when they did not use cannabis. It is important to note, however, that cannabis users reported using cannabis and engaging in exercise nearly every day (*M* = 75% and 81% of survey days, respectively). Daily associations between cannabis use and exercise were not moderated by cannabis condition (*p*s > 0.15).

### 3.3. Cannabis use and diet across the 30-day daily diary study

**Figure 1:**
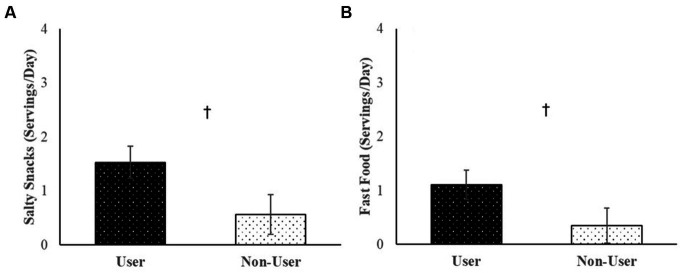
Average servings of **(A)** salty snacks, and **(B)** fast food consumed per day over the 30-day daily diary study by user status.

Across the 30-day daily diary study, participants consumed an average of 3.04 servings of fruits and vegetables (*B* = 3.04, *SE* = 0.25, *p* < 0.001), 1.31 servings of sweet treats (*B* = 1.31, *SE* = 0.21, *p* < 0.001), 1.14 servings of salty snacks (*B* = 1.14, *SE* = 0.26, *p* < 0.001), 0.80 servings of fast food (*B* = 0.80, *SE* = 0.22, *p* = 0.003), and 0.84 servings of sugary drinks (*B* = 0.84, *SE* = 0.19, *p* < 0.001) per day. Although cannabis users reported marginally more servings of salty snacks (*B* = −0.97, *SE* = 0.48, *p* = 0.06) and fast food (*B* = −0.76, *SE* = 0.42, *p* = 0.09) per day relative to non-users (Figure 1), average daily servings of fruits and vegetables (*B* = 0.84, *SE* = 0.62, *p* = 0.17), sweet treats (*B* = −0.46, *SE* = 0.43, *p* = 0.30), and sugary drinks (*B* = −0.43, *SE* = 0.38, *p* = 0.27) did not differ between users and non-users. Additionally, there were no significant differences in daily servings of fruits and vegetables, sweet treats, salty snacks, fast food, or sugary drinks between non-users, users assigned to use THC-dominant products, and users assigned to use products with CBD (*p*s > 0.11).

**Figure 2:**
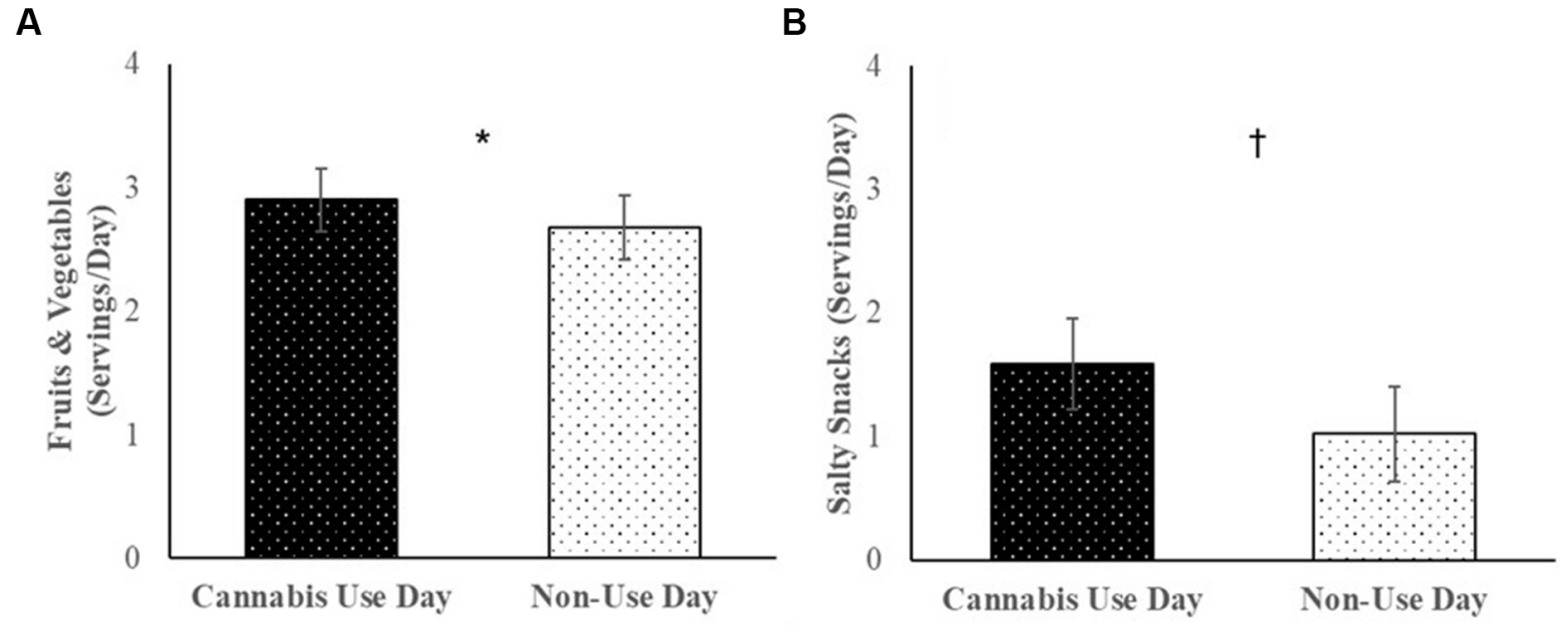
Among cannabis users, average servings of **(A)** fruits and vegetables, and **(B)** salty snacks consumed per day over the 30-day daily diary study on cannabis use days vs. non-use days.

Cannabis users reported consuming more fruits and vegetables (*B* = 0.23, *SE* = 0.08, *p* = 0.01) and marginally more salty snacks (*B* = 0.78, *SE* = 0.38, *p* = 0.07) on days when they used cannabis vs. days when they did not use cannabis (Figure 2). Servings of sweet treats (*B* = 0.49, *SE* = 0.31, *p* = 0.15), fast food (*B* = 0.94, *SE* = 0.43, *p* = 0.26) and sugary drinks (*B* = 0.14, *SE* = 0.29, *p* = 0.64) did not significantly differ between use and non-use days. Daily associations between cannabis use and diet were not moderated by cannabis condition, *p*s > 0.28.

## 4. Discussion

Contrary to hypotheses, there were very few differences in health or health behaviors between individuals who engaged in cannabis use relative to those who did not, either at baseline or longitudinally. Though there was a negative correlation between frequency of cannabis flower use and BMI, such that as cannabis use frequency increased BMI decreased among users, there were no difference in BMI between cannabis users and non-users overall at baseline. Similarly, there were no differences in exercise behavior; users and non-users did not differ in total days of exercise, minutes of moderate exercise, or minutes of vigorous exercise reported over the previous 7 days at baseline. Likelihood of exercising and minutes of exercise per day over the 30-day daily diary period also did not differ between users and non-users, nor did these outcomes differ between cannabis use and non-use days among cannabis using participants.

These results are in contrast to previous findings demonstrating that individuals who use cannabis have lower BMIs than individuals who do not (Smit and Crespo, 2001; Rodondi et al., 2006; Le Strat and Le Foll, 2011; Korn et al., 2018; Ross et al., 2020; York Williams et al., 2020) and that cannabis users engage in more exercise than non-users (Korn et al., 2018; York Williams et al., 2020; Ong et al., 2021; Smith et al., 2021). However, there are a few important differences between the present study and prior work that may explain these discrepancies. One is that definitions of “cannabis user” have varied across studies, with many categorizing cannabis user status based on “ever” using or “any” use over large periods of time rather than comparing current, regular users to those who have not used recently as in the present study. Additionally, prior work has utilized large-scale, epidemiological surveys with thousands of participants making it possible that even small effect sizes are statistically significant. Our sample consisted of 98 individuals such that small, though potentially still clinically meaningful, differences in exercise behavior would not be significant. Alternatively, it is also possible that previously documented cross-sectional associations between cannabis use and these health outcomes have been driven by between-subjects third-variable confounds (e.g., differences in demographic factors between cannabis users and non-users which may be associated with both cannabis use and exercise behavior). It is also important to note that there was a higher proportion of men in the cannabis user (vs. non-user) group, which may have obscured associations between cannabis user group and BMI.

Unlike BMI and exercise, there was some evidence for a relationship between cannabis use and dietary measures. Specifically, baseline findings indicated that frequency of edible use days was correlated with consuming more calories and fat while frequency of cannabis concentrate use days was correlated with consuming more fruits. Further, non-users scored higher on the HEI than users. Across the 30-day daily diary period, users also reported marginally more servings of salty snacks and fast food per day relative to non-users. More fruits/vegetables and marginally more salty snacks were consumed on cannabis use days compared to non-use days among cannabis using participants. Although not significant, users also reported consuming more sugary beverages and sweets treats than non-users, and users reported higher intake of fast food, sugary beverages, and sweet treats on use relative to non-use days. However, these latter findings should be interpreted with caution as the items on sweet treats, salty snacks, fast food, and sugary drinks were only collected for a small portion of participants (*n* = 15) given their late addition to the study. Overall, though, dietary differences between non-users and users parallel previous research findings of associations between cannabis use and greater caloric intake (Smit and Crespo, 2001, Rodondi et al., 2006, Kruger et al., 2019). increased consumption of unhealthy food (Smit and Crespo, 2001; Kruger et al., 2019; Romano et al., 2022), and poorer diet quality (Gelfand and Tangney, 2021), as well as findings that junk food sales increase following initiation of recreational cannabis sales (Baggio and Chong, 2020).

Of note, none of the findings appeared to be dependent on cannabinoid content as there were no significant differences between non-users, users assigned to use THC-dominant products, and users assigned to use products with CBD on any outcome. This finding is surprising given that THC and CBD can differentially affect biological systems and behavioral processes relevant to health (Bhattacharyya et al., 2010; Morgan et al., 2010; Englund et al., 2013; Gibson et al., 2022). However, the lack of findings here may be a function of the observational nature of the study. Specifically, cannabis using participants were allowed to switch from their quasi-randomly assigned product condition to another condition as they wished (i.e., legal restrictions around the study of legal-market cannabis use meant that we could not mandate that they accept their condition assignment). Indeed, several participants who were in the CBD-dominant product condition elected to use products containing some or predominantly THC, and the small number of participants using CBD-dominant products may have thus obscured any differences between a CBD-dominant product vs. THC-dominant products that could have been present. More research is needed to establish whether or not diet and exercise behaviors are truly unaffected by product cannabinoid content.

### 4.1. Study limitations

The fact that participants were able to switch from their quasi-randomly assigned condition is one of several limitations related to federal regulations surrounding cannabis research. Other limitations in this vein include the lack of participant blinding or a standardized dosing procedure. Specifically, state legal-market cannabis products must be accurately labeled with THC and CBD content, thus participants knew what kind of product they purchased and used during the study. Expectancies may have therefore impacted the behaviors assessed in this study. Likewise, we were unable to designate how much or how frequently participants used any product and were also unable to ensure that they only used one type of product throughout the daily diary period. This may have resulted in individuals using different products up to levels of exposure that were comparable (e.g., participants using products with both THC and CBD may have used enough to reach the same level of THC exposure as those using THC-dominant products).

Given the nature of the daily data (i.e., a single survey completed at the end of each day), we were unable to conduct lagged models with our data. Thus, although we were able to determine whether cannabis use, diet, and exercise covaried at a daily level, we were unable to determine the order in which these behaviors occurred each day or whether they occurred together (e.g., whether consumption of salty snacks occurs immediately after cannabis use). Future research in this area should consider implementing ecological momentary assessment (EMA) designs or daily diary studies that collect temporal information.

Additionally, on the daily surveys, participants were not provided with a description of what constitutes a food serving which may have led to variability in how participants responded to the daily diet questions. As individuals often find it difficult to judge appropriate portion sizes [e.g., underestimating what constitutes a serving of fruits or vegetables or overestimating what constitutes a serving of fast food (Hebert et al., 2008; Almiron-Roig et al., 2013)], future studies should provide standardized examples of serving sizes for each dietary category that is assessed (e.g., Gardiner and Bryan, 2017). Future studies should also consider including more detailed questions about exercise behavior. For instance, although minutes of exercise per day did not differ between users and non-users or between cannabis use and non-use days, it is possible that there were differences in the type (e.g., aerobic vs. anaerobic) and intensity (e.g., high vs. low intensity) of exercise that we were unable to detect given the single item assessing exercise on the daily surveys (although it is worth noting that there were no differences in exercise intensity at baseline between users and non-users).

Outside of federal regulation limitations, another limitation was the lack of diversity in the sample itself. The participants in this study were mostly affluent White individuals who were highly active and healthy. Indeed, the average BMI was within healthy limits, and baseline reports of exercise were well above the standard guidelines (e.g., 175 min of vigorous activity per week as compared to national recommendations advising 75 min per week; USDA, 2022). Further, participants exercised almost all of the 30 days they were surveyed. As a result, it is possible that differences in exercise behavior by cannabis user status – as well as use vs. non-use days – may have been masked by ceiling effects. Future research is needed to determine whether findings generalize to populations facing greater barriers to exercise engagement, including older adults and individuals with overweight/obesity. Relatedly, cannabis using participants reported using cannabis either every day or almost every day. This small amount of variability across measures might have made it difficult to detect differences between users and non-users or differences between use vs. non-use days within cannabis using participants, underscoring the need for studies with naïve or infrequent cannabis users.

A few other limitations include that all participants in the cannabis user group were regular users and, for the purposes of the study, were instructed to use flower products. Thus, these findings may not generalize to other types of cannabis products (e.g., concentrates) or to individuals who use cannabis less frequently. Lastly, in line with previous findings that men are more likely to use cannabis than women (Cuttler et al., 2016), there were significantly more men in the cannabis user group (although it should be noted that findings held when controlling for participant gender).

### 4.2. Study strengths

Despite these limitations, the study had several notable strengths. These include our focus on the association between *ad libitum* use of commercially available products and health metrics and behaviors, the use of quasi-randomization to investigate differences in THC and CBD content on these measures, and the daily diary methodology. All of these facilitated greater external validity (i.e., examination of the products individuals normally use in environments they normally use them in), while also affording us some measure of internal validity (i.e., although limited, greater manipulation of cannabinoid content and the ability to match cannabis use and behavior in time using daily assessments that minimize recall bias). Continued research utilizing methodologies that allow for exploration of how products that individuals commonly use affect health and health behavior is nevertheless critical. This is especially relevant given the increasing variety of cannabis products that individuals have access to in state regulated markets. A final strength is that our study utilized a more nuanced assessment of cannabis use and user status rather than simply dichotomizing “ever user” and “never user” (Cousijn et al., 2018; Geissler et al., 2020; Bidwell et al., 2021).

### 4.3. Conclusion

In sum, our data do not support concerns that cannabis use may lead to higher levels of physical inactivity as there were no differences in exercise behaviors between users and non-users nor was there a difference in exercise between cannabis use and non-use days among users. On the other hand, there was some evidence for a relationship between cannabis use and diet whereby, in general, cannabis users reported less healthy eating than non-users. Interestingly, despite these dietary differences, BMI did not differ between users and non-users, perhaps owing to both groups’ high level of physical activity. Still, these findings underscore the need for additional research aimed at understanding how cannabis may impact both behavioral and non-behavioral pathways to obesity and chronic disease (e.g., metabolism, inflammation, etc.). Expanded research in these domains, and particularly with more diverse populations and a greater variety of cannabis products, is needed to increase our understanding of the harms and benefits of legal-market cannabis use.

## Data availability statement

The raw data supporting the conclusions of this article will be made available by the authors, without undue reservation.

## Ethics statement

This study was reviewed and approved by the University of Colorado Boulder Institutional Review Board. The participants provided their written informed consent to participate in this study.

## Author contributions

AB was responsible for study design and for overseeing all aspects of study execution and manuscript preparation. LG was responsible for conceptualizing and performing the data analysis. GG contributed to data cleaning and analysis. LG, CS, and GG led the interpretation and write-up of findings. All authors critically reviewed content and approved the final version for publication.

## Funding

This work was supported by the National Institute on Drug Abuse (1R01DA050515 to AB). LG is funded by a National Science Foundation Graduate Research Fellowship (DGE 1650115).

## Conflict of interest

The authors declare that the research was conducted in the absence of any commercial or financial relationships that could be construed as a potential conflict of interest.

## Publisher’s note

All claims expressed in this article are solely those of the authors and do not necessarily represent those of their affiliated organizations, or those of the publisher, the editors and the reviewers. Any product that may be evaluated in this article, or claim that may be made by its manufacturer, is not guaranteed or endorsed by the publisher.

## Footnotes

^1^ https://www.r-project.org/

## References

[ref1] Almiron-Roig E. Solis-Trapala I. Dodd J. Jebb S. A. (2013). Estimating food portions. Influence of unit number, meal type and energy density. Appetite 71, 95–103. doi: 10.1016/j.appet.2013.07.012, PMID: 23932948 PMC3857597

[ref2] American College of Sports Medicine. ACSM’s guidelines for exercise testing and prescription. (2021). Available at: https://www.acsm.org/education-resources/books/guidelines-exercise-testing-prescription (accessed May 4, 2023). 10.1249/JSR.0b013e31829a68cf 23851406

[ref3] Baggio M. Chong A. (2020). Recreational marijuana laws and junk food consumption. Econ. Hum. Biol. 39, 100922–100913. doi: 10.1016/j.ehb.2020.100922, PMID: 32992092

[ref4] Bates D. Mächler M. Bolker B. Walker S. (2015). Fitting linear mixed-effects models using lme 4. J. Stat. Softw. 67, 1–48.

[ref5] Bhattacharyya S. Morrison P. D. Fusar-Poli P. Martin-Santos R. Borgwardt S. Winton-Brown T.. (2010). Opposite effects of Δ-9-tetrahydrocannabinol and cannabidiol on human brain function and psychopathology. Neuropsychopharmacology 35, 764–774. doi: 10.1038/npp.2009.184, PMID: 19924114 PMC3055598

[ref6] Bidwell L. C. Martin-Willett R. Karoly H. C. (2021). Advancing the science on cannabis concentrates and behavioural health. Drug Alcohol Rev. 40, 900–913. doi: 10.1111/dar.13281, PMID: 33783029 PMC9878551

[ref7] Blair S. N. Haskell W. L. Ho P. Paffenbarger R. S. Jr. Vranizan K. M. Farquhar J. W.. (1985). Assessment of habitual physical activity by a seven-day recall in a community survey and controlled experiments. Am. J. Epidemiol. 122, 794–804. doi: 10.1093/oxfordjournals.aje.a114163 3876763

[ref8] Centers for Disease Control and Prevention. Get the facts: added sugars. (2021). Available at: https://www.cdc.gov/nutrition/data-statistics/added-sugars.html#:~:text=Americans%20should%20limit%20their%20added%20sugars&text=Americans%202%20years%20and%20older,sugars%20(about%2012%20teaspoons) (accessed May 4, 2023).

[ref9] Centers for Disease Control and Prevention. Defining adult overweight & obesity. (2022). Available at: https://www.cdc.gov/obesity/basics/adult-defining.html (accessed May 4, 2023).

[ref10] Centers for Disease Control and Prevention. Adults meeting fruit and vegetable intake recommendations—United States, 2019. (2022). Available at: https://www.cdc.gov/mmwr/volumes/71/wr/mm7101a1.htm#:~:text=Adults%20should%20consume%201.5%E2%80%932cup%2Dequivalents%20of%20vegetables%20daily (accessed May 4, 2023).

[ref11] Cousijn J. Núñez A. E. Filbey F. M. (2018). Time to acknowledge the mixed effects of cannabis on health: a summary and critical review of the NASEM 2017 report on the health effects of cannabis and cannabinoids. Addiction 113, 958–966. doi: 10.1111/add.14084, PMID: 29271031 PMC9520128

[ref12] Cuttler C. Mischley L. K. Sexton M. (2016). Sex differences in cannabis use and effects: a cross-sectional survey of cannabis users. Cann. Can. Res. 1, 166–175. doi: 10.1089/can.2016.0010, PMID: 28861492 PMC5576608

[ref13] Defense Information Systems Agency. Marijuana legality by state. (2022). Available at: https://disa.com/maps/marijuana-legality-by-state (accessed May 4, 2023).

[ref14] Englund A. Morrison P. D. Nottage J. Hague D. Kane F. Bonaccorso S.. (2013). Cannabidiol inhibits THC-elicited paranoid symptoms and hippocampal-dependent memory impairment. J. Psychopharmacol. 27, 19–27. doi: 10.1177/0269881112460109, PMID: 23042808

[ref15] Gardiner C. K. Bryan A. D. (2017). Monetary incentive interventions can enhance psychological factors related to fruit and vegetable consumption. Ann. Behav. Med. 51, 599–609. doi: 10.1007/s12160-017-9882-4, PMID: 28176150

[ref16] Geissler K. H. Kaizer K. Johnson J. K. Doonan S. M. Whitehill J. M. (2020). Evaluation of availability of survey data about cannabis use. JAMA Netw. Open 3:3. doi: 10.1001/jamanetworkopen.2020.6039 PMC7287570 32520358

[ref17] Gelfand A. R. Tangney C. C. (2021). Dietary quality differs among cannabis use groups: data from the National Health and nutrition examination survey 2005–16. Public Health Nutr. 24, 3419–3427. doi: 10.1017/S1368980020001846, PMID: 32698932 PMC10195383

[ref18] Gibson L. P. Karoly H. C. Ellingson J. M. Klawitter J. Sempio C. Squeri J. E.. (2022). Effects of cannabidiol in cannabis flower: implications for harm reduction. Addict. Biol. 27:27. doi: 10.1111/adb.13092 PMC9357513 34467598

[ref19] Hagerty S. L. Williams S. L. Mittal V. A. Hutchison K. E. (2015). The cannabis conundrum: thinking outside the THC box. J. Clin. Pharmacol. 55, 839–841. doi: 10.1002/jcph.511, PMID: 25855064

[ref20] Harris P. A. Taylor R. Thielke R. Payne J. Gonzalez N. Conde J. G. (2009). Research electronic data capture (REDCap)—a metadata-driven methodology and workflow process for providing translational research informatics support. J. Biomed. Inform. 42, 377–381. doi: 10.1016/j.jbi.2008.08.010, PMID: 18929686 PMC2700030

[ref21] Hayatbakhsh M. R. O'Callaghan M. J. Mamun A. A. Williams G. M. Clavarino A. Najman J. M. (2010). Cannabis use and obesity and young adults. Am. J. Drug Alcohol Abuse 36, 350–356. doi: 10.3109/00952990.2010.500438, PMID: 20936991

[ref22] Hebert J. R. Hurley T. G. Peterson K. E. Resnicow K. Thompson F. E. Yaroch A. L.. (2008). Social desirability trait influences on self-reported dietary measures among diverse participants in a multicenter multiple risk factor trial. J. Nutr. 138, 226S–234S. doi: 10.1093/jn/138.1.226S, PMID: 18156429

[ref23] Hutchison K. E. Bidwell L. C. Ellingson J. M. Bryan A. D. (2019). Cannabis and health research: rapid progress requires innovative research designs. Value Health 22, 1289–1294. doi: 10.1016/j.jval.2019.05.005, PMID: 31708066

[ref24] Imtiaz S. Rehm J. (2018). The relationship between cannabis use and diabetes: results from the National Epidemiologic Survey on alcohol and related conditions III. Drug Alcohol Rev. 37, 897–902. doi: 10.1111/dar.12867, PMID: 30288813

[ref25] Korn L. Haynie D. L. Luk J. W. Simons-Morton B. G. (2018). Prospective associations between cannabis use and negative and positive health and social measures among emerging adults. Int. J. Drug Policy 58, 55–63. doi: 10.1016/j.drugpo.2018.05.003, PMID: 29807247 PMC11349058

[ref26] Kruger J. S. Blavos A. Castor T. S. Wotring A. J. Wagner-Greene V. R. Glassman T.. (2019). Manipulation checking the munchies: validating self-reported dietary behaviors during cannabis intoxication. Hum. Ethol. 34, 10–16. doi: 10.22330/he/34/010-016

[ref27] Le Strat Y. Le Foll B. (2011). Obesity and cannabis use: results from 2 representative national surveys. Am. J. Epidemiol. 174, 929–933. doi: 10.1093/aje/kwr200, PMID: 21868374

[ref28] Martin-Willett R. Helmuth T. Abraha M. Bryan A. D. Hitchcock L. Lee K.. (2020). Validation of a multisubstance online timeline Followback assessment. Brain Behav. 10:10. doi: 10.1002/brb3.1486 PMC6955818 31793226

[ref29] Morgan C. J. Freeman T. P. Schafer G. L. Curran H. V. (2010). Cannabidiol attenuates the appetitive effects of Δ9-tetrahydrocannabinol in humans smoking their chosen cannabis. Neuropsychopharmacology 35, 1879–1885. doi: 10.1038/npp.2010.58, PMID: 20428110 PMC2906701

[ref30] O’Neill B. J. (2020). Effect of low-carbohydrate diets on cardiometabolic risk, insulin resistance, and metabolic syndrome. Curr. Opin. Endocrinol. Diabetes Obes. 27, 301–307. doi: 10.1097/MED.0000000000000569, PMID: 32773574

[ref31] Ong L. Q. Bellettiere J. Alvarado C. Chavez P. Berardi V. (2021). Cannabis use, sedentary behavior, and physical activity in a nationally representative sample of US adults. Harm Reduct. J. 18, 1–11. doi: 10.1186/s12954-021-00496-2 33926458 PMC8086340

[ref32] Rodondi N. Pletcher M. J. Liu K. Hulley S. B. Sidney S. (2006). Marijuana use, diet, body mass index, and cardiovascular risk factors (from the CARDIA study). Am. J. Cardiol. 98, 478–484. doi: 10.1016/j.amjcard.2006.03.024, PMID: 16893701

[ref33] Romano E. Ma R. Vancampfort D. Smith L. Firth J. Solmi M.. (2022). The association of cannabis use with fast-food consumption, overweight, and obesity among adolescents aged 12-15 years from 28 countries. J. Subst. Abus. 31, 1–10. doi: 10.1080/14659891.2022.2114388

[ref34] Ross J. M. Pacheco-Colón I. Hawes S. W. Gonzalez R. (2020). Bidirectional longitudinal associations between cannabis use and body mass index among adolescents. Can. Can. Res. 5, 81–88. doi: 10.1089/can.2019.0091, PMID: 32322679 PMC7173669

[ref9001] Saunders J. B. Aasland O. G. Babor T. F. de la Fuente J. R. Grant M. (1993). Development of the Alcohol Use Disorders Identification Test (AUDIT): WHO Collaborative Project on Early Detection of Persons with Harmful Alcohol Consumption-II. Addiction 88, 791–804. 8329970 10.1111/j.1360-0443.1993.tb02093.x

[ref35] Schakel S. F. Buzzard I. M. Gebhardt S. E. (1997). Procedures for estimating nutrient values for food composition databases. J. Food Comp. Anal. 10, 102–114. doi: 10.1006/jfca.1997.0527, PMID: 36074559

[ref36] Smit E. Crespo C. J. (2001). Dietary intake and nutritional status of US adult marijuana users: results from the third National Health and nutrition examination survey. Public Health Nutr. 4, 781–786. doi: 10.1079/PHN2000114, PMID: 11415485

[ref37] Smith L. Sherratt F. Barnett Y. Cao C. Tully M. A. Koyanagi A.. (2021). Physical activity, sedentary behaviour and cannabis use in 15, 822 US adults: cross-sectional analyses from NHANES. Public Health 193, 76–82. doi: 10.1016/j.puhe.2021.01.018, PMID: 33743217

[ref38] Sobell L. C. Sobell M. B. (1992). Timeline follow-back: a technique for assessing self-reported alcohol consumption. Psychosoc. Biochem. Methods, 41–72. doi: 10.1007/978-1-4612-0357-5_3

[ref39] Substance Abuse and Mental Health Services Administration. Key substance use and mental health indicators in the United States: Results from the 2018 National Survey on drug use and health. (2019). Available at: https://www.samhsa.gov/data/sites/default/files/cbhsq-reports/NSDUHNationalFindingsReport2018/NSDUHNationalFindingsReport2018.pdf (accessed May 4, 2023).

[ref40] Substance Abuse and Mental Health Services Administration. 2021 NSDUH Detailed Tables. (2021). Available at: https://www.samhsa.gov/data/report/2021-nsduh-detailed-tables (accessed May 4, 2023).

[ref41] USDA. (2022). Healthy Eating Index Available at: https://www.fns.usda.gov/hei-scores-americans (accessed May 4, 2023).

[ref42] Vidot D. C. Bispo J. B. Hlaing W. M. Prado G. Messiah S. E. (2017). Moderate and vigorous physical activity patterns among marijuana users: results from the 2007–2014 National Health and nutrition examination surveys. Drug Alcohol Depend. 178, 43–48. doi: 10.1016/j.drugalcdep.2017.05.004, PMID: 28641129

[ref43] West A. B. Bittel K. M. Russell M. A. Evans M. B. Mama S. K. Conroy D. E. (2020). A systematic review of physical activity, sedentary behavior, and substance use in adolescents and emerging adults. Transl. Behav. Med. 10, 1155–1167. doi: 10.1093/tbm/ibaa008, PMID: 33044536 PMC7549408

[ref44] York Williams S. L. Gibson L. P. Gust C. J. Giordano G. Hutchison K. E. Bryan A. D. (2020). Exercise intervention outcomes with Cannabis users and nonusers aged 60 and older. Am. J. Health Behav. 44, 420–431. doi: 10.5993/AJHB.44.4.5, PMID: 32553024 PMC12240158

[ref45] Yuan X. Wang J. Yang S. Gao M. Cao L. Li X.. (2020). Effect of the ketogenic diet on glycemic control, insulin resistance, and lipid metabolism in patients with T2DM: a systematic review and meta-analysis. Nutr. Diabetes 10:10. doi: 10.1038/s41387-020-00142-z 33257645 PMC7705738

