# Supplementary material for: A daily diary investigation of cannabis use and its diet and exercise correlates
Source: Front Psychol. 2023 Aug 4;14:1217144. doi: 10.3389/fpsyg.2023.1217144 (PMC10436564; doi:10.3389/fpsyg.2023.1217144)
Supplement: Supplementary file 1 [file Table_1.pdf]

Supplementary Materials

Supplementary Table 1. Bivariate correlations between baseline BMI, exercise, diet, and cannabis use variables among both cannabis users and non-users (*N* = 98).

	1	2	3	4	5	6	7	8	9	10	11	12	13	14	15	16
1. BMI																
2. Total Days of Exercise	.09															
3. Minutes of Moderate Exercise	.05	**.34**														
4. Minutes of Hard Exercise	-.10	**.25**	-.09													
5. Total Calories Consumed	.00	**.23**	.13	.13												
6. Carbohydrates Consumed	-.10	.17	.12	-.17	**.76**											
7. Fat Consumed	.04	.18	.10	**.27**	**.88**	**.39**										
8. Sodium Consumed	.10	**.25**	.08	**.33**	**.79**	**.36**	**.76**									
9. Protein Consumed	.05	.05	.09	.18	**.66**	**.42**	**.63**	**.56**								
10. HEI Total Score	-.14	.16	.02	-.09	.08	.14	.03	.06	-.19							
11. HEI Total Fruit	-.10	-.07	-.03	-.17	-.15	.06	**-.25**	-.15	**-.31**	**.43**						
12. HEI Total Vegetable	.00	.08	-.07	-.01	-.02	-.04	.02	.00	.07	**.27**	-.16					
13. HEI Added Sugar Score	.13	.03	-.03	.19	.04	**-.25**	**.22**	**.26**	.13	**.33**	-.03	**.41**				
14. Cannabis Flower Use Days	-.17	-.04	.05	-.01	-.18	-.14	-.15	-.14	-.07	-.02	-.06	.07	.05			
15. Cannabis Edible Use Days	.13	-.03	-.04	.17	.16	.00	**.29**	.12	.11	-.05	-.05	.16	.10	.06		
16. Cannabis Concentrate Use Days	.15	-.14	.01	.05	-.06	-.12	-.02	.02	.01	-.08	.17	-.11	-.12	**-.22**	.14	
17. Age at First Cannabis Use	-.05	.18	**-.32**	.13	.05	.02	.01	.15	.11	.17	-.01	.05	.09	**-.24**	-.13	-.02
